# Neurosarcoidosis: A Case Report

**DOI:** 10.7759/cureus.24501

**Published:** 2022-04-26

**Authors:** Margarida L Nascimento, Rui Casanova, Filipa Ramalho Rocha, Filipa Malheiro, Pedro Araújo

**Affiliations:** 1 Internal Medicine Department, Hospital Da Luz Lisboa, Lisbon, PRT; 2 Intensive Care Unit, Hospital Da Luz Lisboa, Lisbon, PRT; 3 Internal Medicine Department, Hospital da Luz Lisboa, Lisbon, PRT; 4 Otolaryngology Department, Hospital da Luz Lisboa, Lisbon, PRT

**Keywords:** noncaseating granulomas, granulomatous diseases, extrapulmonary sarcoidosis, sarcoidosis, neurosarcoidosis

## Abstract

Sarcoidosis is a multi-organ granulomatous disease of unknown etiology. Neurological involvement in sarcoidosis is uncommon but cranial mononeuropathies, especially involving the VII and VIII cranial nerves, are highly suggestive of neurosarcoidosis. We report the case of a 54-year-old woman who presented with fever, night sweats, weight loss, polyarthralgia, and bilateral hearing loss. Mediastinal and hilar lymphadenopathies with hypercaptation on positive emission tomography (PET) scans were present. Low-dose steroids were ineffective. She then developed bilateral anterior uveitis and right-sided peripheral facial palsy. Head magnetic resonance imaging (MRI) showed inflammatory involvement of the right cochlea, geniculate ganglion, and bilateral vestibulocochlear bundle. Cerebrospinal fluid analysis was compatible with aseptic meningitis. Excisional biopsy of mediastinal lymph nodes confirmed the presence of noncaseating granulomas. The diagnosis of systemic sarcoidosis with serious neurological involvement was made and treatment with high-dose steroids led to significant clinical improvement. Sarcoidosis remains a diagnosis of exclusion based on supportive clinical, radiological, and histological findings. This case highlights the challenge it was to diagnose this disorder. Neurologic involvement in sarcoidosis is relatively uncommon and has an unpredictable clinical course and prognosis.

## Introduction

Sarcoidosis is a multi-organ granulomatous disease of unknown etiology and is characterized pathologically by multiple noncaseating granulomas in the absence of a defined trigger [1]. It mostly affects middle-aged patients and there is a slight female predominance. The most commonly affected organs are the lungs and intrathoracic lymph nodes [2]. The clinical and radiological diagnosis of sarcoidosis is not standardized but is based on three major criteria: a compatible clinical presentation, findings of non-necrotizing granulomatous inflammation, and the exclusion of alternative causes [3].

Neurological involvement in sarcoidosis is relatively uncommon, with a reported prevalence of 3-10%. Any part of the nervous system can be affected, with the cranial nerves, meninges, and brain parenchyma being the most commonly involved [2]. Cranial nerves II, VII, and VIII are the most commonly affected. In over 90% of cases of neurosarcoidosis (NS), systemic manifestations are also observed [2]. However, in almost 50% of patients with suspected NS, the neurologic symptoms represent the first defining manifestation of sarcoidosis [4].

Despite numerous publications on NS, no consensual definition exists. Regarding the diagnosis, the difficulty of obtaining neural tissue for histologic analysis presents a major challenge. As such, NS is typically assumed when a patient has clinical evidence of central or peripheral nervous system inflammation consistent with NS and pathologic proof of sarcoidosis in another organ system [4].

## Case presentation

A 54-year-old woman with no relevant medical history presented to the outpatient clinic with complaints of symmetrical polyarthralgia, involving the small joints of her hands, with edema and morning stiffness, bilateral hearing loss, low-grade fever, night sweats, and involuntary weight loss over the last month. Initial evaluation performed in another institution showed a rheumatoid factor of 133 UI/mL and negative levels of antinuclear, anti-neutrophil cytoplasmic, double-stranded deoxyribonucleic acid, extractable nuclear antigen, and cyclic citrullinated peptide antibodies. She was started on deflazacort 6 mg daily with no clinical improvement after three weeks of treatment.

**Figure 1:**
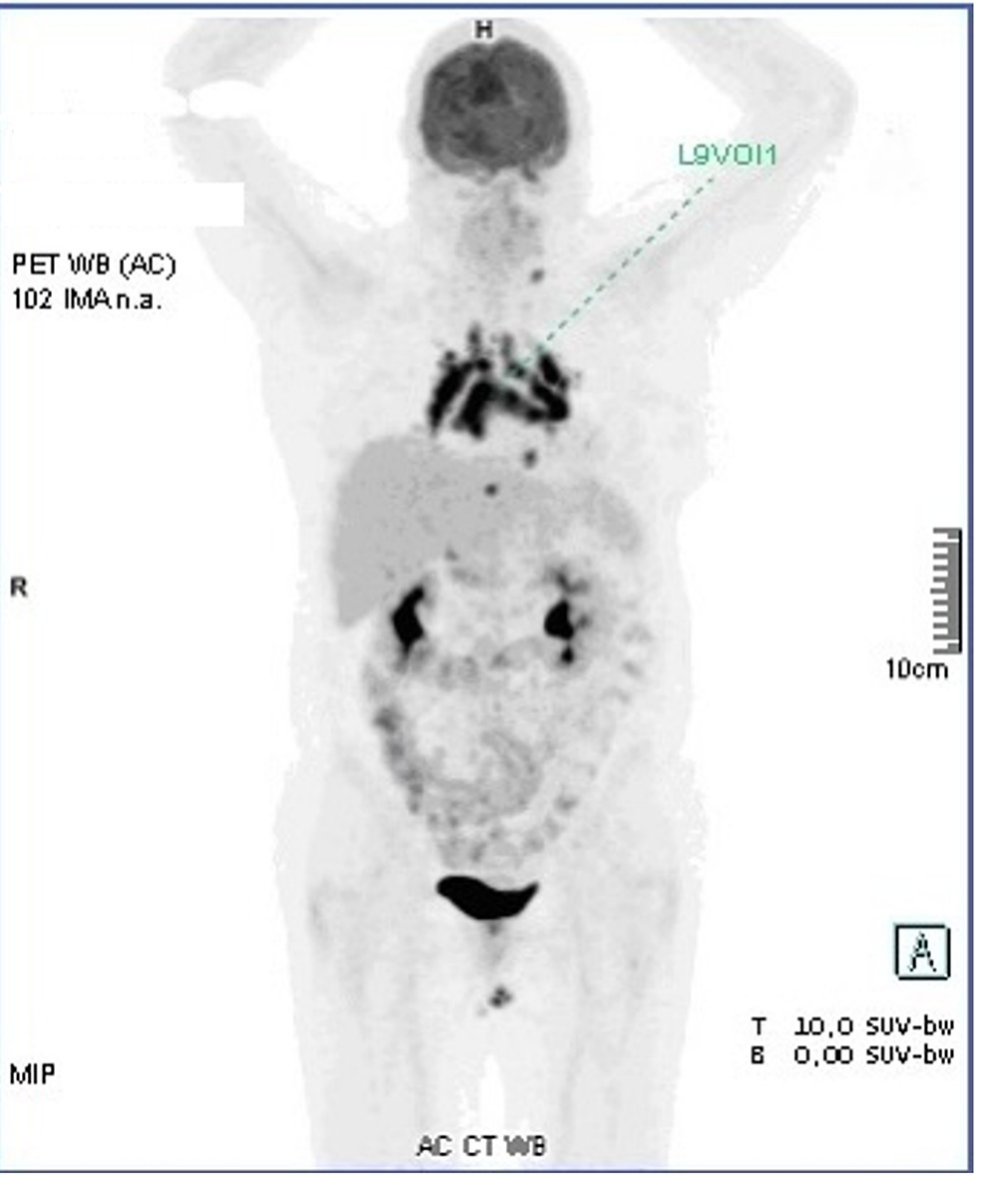
18F-FDG PET-CT scan. FDG: fluorodeoxyglucose; PET: positron emission tomography Maximum intensity projection (MIP) whole-body image showing increased uptake in multiple lymph nodes (mediastinal, bilateral hilar, left supraclavicular and pre-aortic). The SUVmax was 14.89.

The patient was referred to our hospital, where chest computed tomography was performed that identified multiple and bulky mediastinal and hilar adenopathies. Blood tests revealed an erythrocyte sedimentation rate of 70 mm/h and an angiotensin-converting enzyme level of 124.7 UI/L. Serological tests for human immunodeficiency virus (HIV), cytomegalovirus, toxoplasmosis, and venereal disease research laboratory test (VDRL) were negative. Serum protein electrophoresis (SPEP) was normal. Endobronchial ultrasonography (EBUS) showed no significant macroscopic changes and histology of transbronchial needle aspiration (TBNA) on 7 and 11 L lymph nodes stations were inconclusive. A whole-body positive emission tomography (PET) scan documented voluminous hypermetabolic lymphadenopathies predominantly in the pulmonary hilum and all mediastinal compartments with a maximum standardized uptake value (SUVmax) of 14.89 and a left supraclavicular and two adjacent to descending aorta lymphadenopathies with SUVmax of 8.27 (Figure 1).

**Figure 2:**
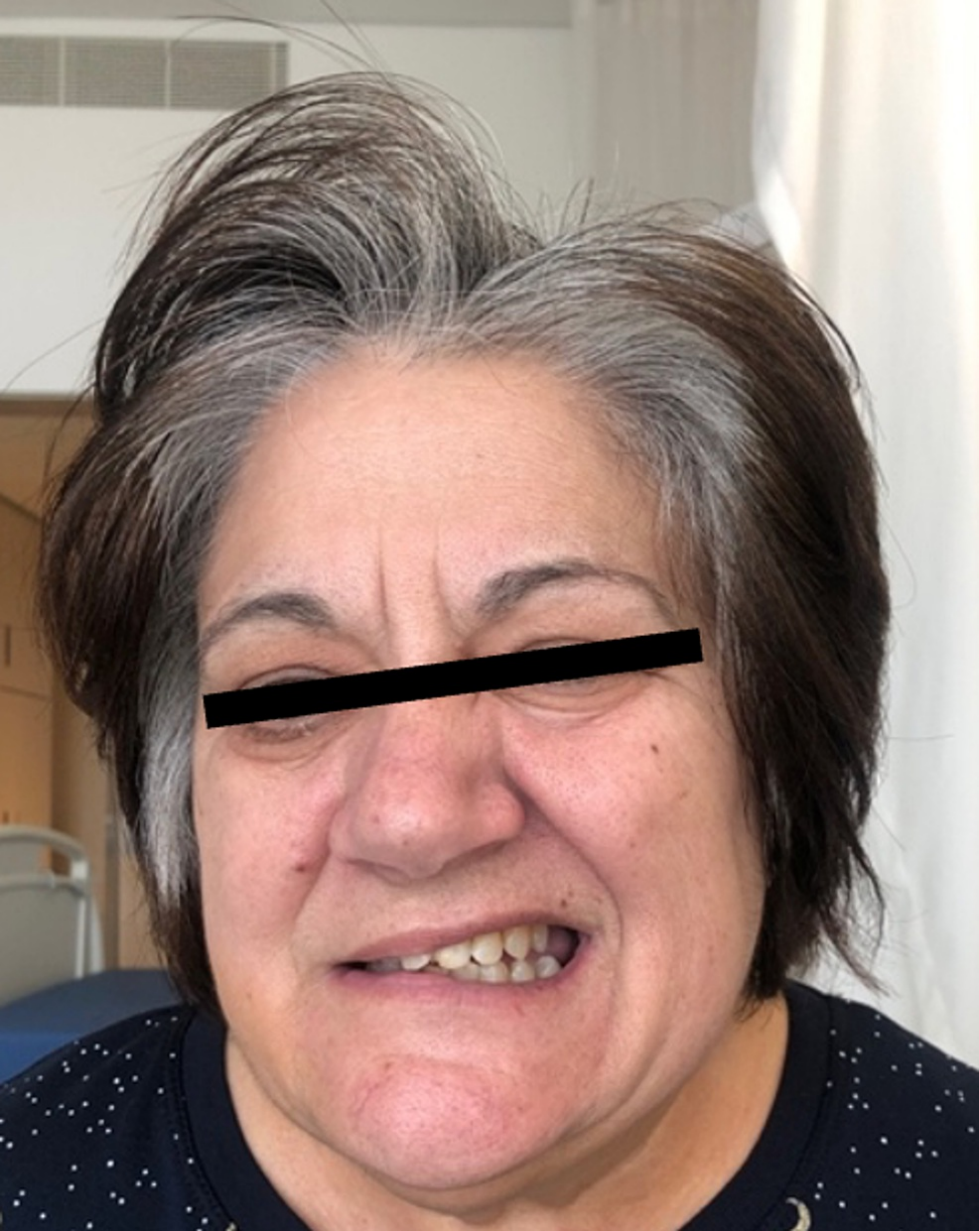
Right-sided peripheral facial palsy.

During the process of investigation, the patient complained of painful red eye, first right-sided and then bilaterally, leading to the diagnosis of bilateral anterior uveitis after ophthalmology consultation. There was a remarkable improvement in the topic of prednisolone. Additionally, acute onset of right-sided peripheral facial palsy (Figure 2), grade 3 of the House-Brackmann grading scale, arose along with a sudden increase in the bilateral hearing loss and positional vertigo.

**Figure 3:**
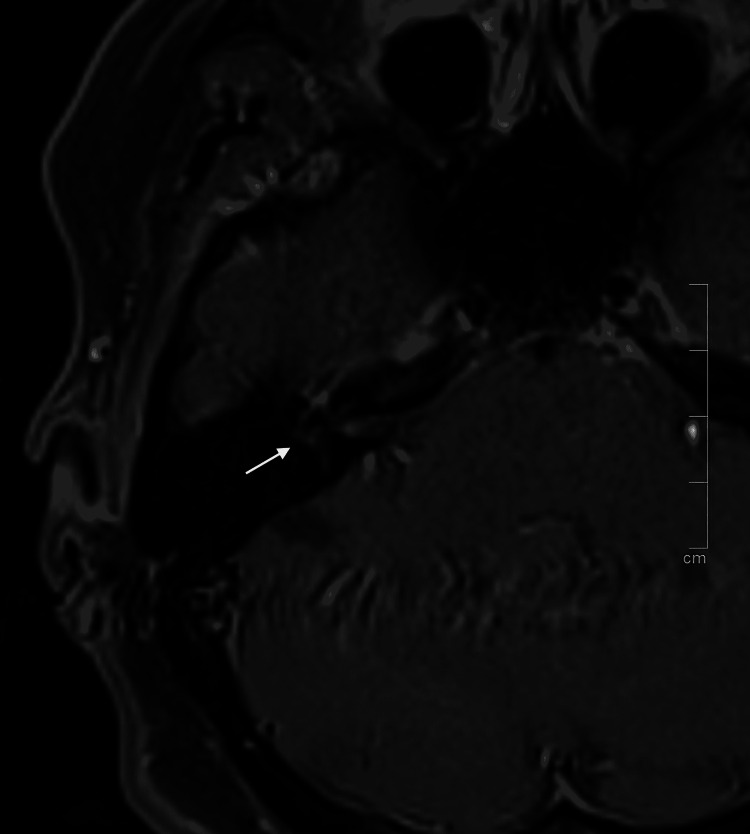
Head magnetic resonance imaging. T1 hyperintensity after gadolinium enhancement of the right-sided cochlear basal turn and geniculate ganglion and enhanced signal of vestibulocochlear bundle bilaterally (white arrow).

Head magnetic resonance imaging (MRI) documented T1 hyperintensity after gadolinium enhancement of the right-sided cochlear basal turn and geniculate ganglion, and enhanced signal of vestibulocochlear bundle bilaterally, greater on the right side, suggesting inflammatory/granulomatous involvement of the cranial nerves, with no leptomeningeal enhancement (Figure 3). Cerebral spinal fluid (CSF) analysis revealed protein levels of 89 mg/dL, glucose levels of 83 mg/dL, and 46 mononuclear cells/µL. CSF cytology revealed the presence of lymphocytes and histiocytes with no malignant cells. Bacterial, mycobacterial, and fungal cultures and also serological tests for Borrelia burgdorferi and VDRL were negative. The otorhinolaryngology consultation concluded the diagnosis of a right ear benign paroxysmal positional vertigo (BPPV) with no lesion of the vestibulo-ocular reflex by video head impulse test (vHIT) and severe bilateral sensorineural hearing loss.

**Figure 4:**
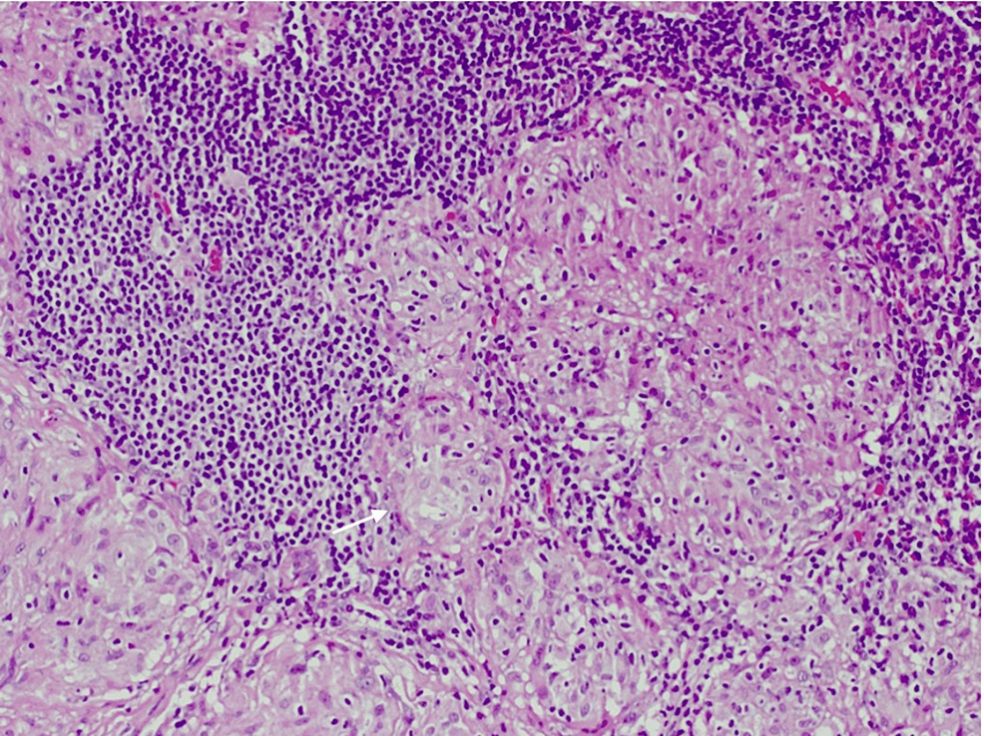
Thoracic lymph nodes histopathological examination. Hematoxylin and eosin stain at 100× magnification, showing lymph nodes almost fully replaced by noncaseating granulomas (white arrow).

The patient underwent video-assisted thoracoscopic surgery (VATS) with lymphadenectomy. Histopathological examination showed lymph nodes almost fully replaced by epithelioid coalescing regular granulomas, with no central necrosis (Figure 4).

The diagnosis of systemic sarcoidosis with remarkable neurological involvement (facial and vestibulocochlear nerves, and laboratory evidence of aseptic meningitis) was made and treatment with prednisolone 1 mg/kg of body weight per day was started. After three months of therapy with slow tapering, systemic symptoms, facial palsy, and head MRI findings resolved. At a nine-month follow-up, vertigo was remarkably better after a successful canalith repositioning procedure, with no improvement of the hearing loss. Unfortunately, the patient lost to follow-up.

## Discussion

Sarcoidosis is a systemic disease with a heterogeneous clinical presentation. For diagnosis, the typical clinical-radiological picture should be supported by the histologic confirmation of noncaseating granulomas [1]. It’s important to rule out other etiologies of granuloma formation such as tuberculosis, fungal infection, foreign body reaction, autoimmune diseases, and drug-allergy [2], and cultures must be negative, as happened in our case. The most commonly affected organs in sarcoidosis are the lungs and intrathoracic lymph nodes (over 90% of patients) and ocular sarcoidosis, following skin involvement, is the second most common extrathoracic manifestation of this disease [2]. Our patient had pulmonary, ocular, and joint involvement.

Neurological involvement in sarcoidosis is uncommon, but every part of the nervous system can be affected, which is what makes differential diagnosis difficult. Cranial mononeuropathies, especially involving the cranial nerves VII and VIII, in the right systemic context are highly suggestive. Other manifestations are papillary edema, aseptic meningitis, hydrocephalus, intracerebral lesions, seizures, or psychiatric symptoms [1].

A major challenge regarding NS is the difficulty of obtaining neural tissue for histologic and microbiologic analyses [4]. The diagnosis is based on neuroimaging studies, particularly MRI [1]. NS can be assumed in patients with confirmed sarcoid involvement of other organs and suggestive neurological symptoms. In our clinical case, we confirmed the diagnosis of sarcoidosis by finding non-necrotizing granulomas in intrathoracic lymph nodes. Although the diagnosis of pulmonary involvement was straightforward, the initial presentation and clinical course with neurological symptoms were quite remarkable and the differential diagnosis was difficult.

NS almost always requires high-dose steroid treatment because spontaneous remission is unlikely and damage associated with the inflammatory lesion can cause permanent neurologic deficit [4]. Our patient evolved with progressive disabling symptoms and organ-threatening disease for which treatment was indicated. The prognosis of facial neuropathy is good, with complete recovery seen in about 90% of patients after treatment with glucocorticoids [2]. The significant improvement seen after therapy highlights the importance of awareness toward this clinical entity in order to start treatment as early as possible.

## Conclusions

Sarcoidosis remains a diagnosis of exclusion based on supportive clinical, radiological, and histological findings. Diverse disease presentations and lack of specificity of diagnostic tests contribute frequently to diagnostic uncertainty. Neurologic involvement in sarcoidosis is relatively uncommon and has an unpredictable clinical course and prognosis. This case highlights the challenge it was to diagnose this disorder.
